# The co-occurrence of *Toxocara* ocular and visceral larva migrans syndrome: a case series

**DOI:** 10.1186/1757-1626-2-6881

**Published:** 2009-05-11

**Authors:** Małgorzata Paul, Jerzy Stefaniak, Hanna Twardosz-Pawlik, Krystyna Pecold

**Affiliations:** 1 Department and Clinic of Tropical and Parasitic Diseases, University of Medical SciencesPrzybyszewskiego 49, 60-355 PoznańPoland; 2 Department of Ophthalmology, University of Medical SciencesDługa 1/2, 61-848 PoznańPoland

## Abstract

**Introduction:**

Ocular toxocarosis associated with high peripheral eosinophilia and together with systemic signs of visceral damage has been reported sporadically. Eye infections caused by numerous migrating larvae of *Toxocara* parasites, probably due to re-invasion or delayed reactivation, and leading to a progressive loss of vision is relatively rare. We report three atypical cases of toxocarosis with the co-existence of ocular larva migrans syndrome and generalized signs of *Toxocara* infection in schoolboys.

**Case presentation:**

Two children aged 8 and 14 years respectively, with symptomatic ocular and visceral larva migrans syndromes, and one 16-year-old adolescent with chronic multifocal eye invasion, characterized by severe granulomatous retinochoroiditis with unilateral blindness, chronic abdominal pain and generalized synthesis of total immunoglobulin E antibody are described. The three patients, heavily infected with *Toxocara species* were boys of Polish origin. Ocular location of the parasite was confirmed by the detection of intraocular synthesis of specific anti-*Toxocara* immunoglobulin G antibody in aqueous humour samples from the affected eyes. Immunological parameters of tissue eosinophilia, allergy or hypersensitivity reactions to the presence of the migrating *Toxocara* parasites were analysed. Irreversible eye complications were observed in the patients with high level of exposure to *Toxocara species* in a contaminated environment, with a suggestion of possible re-activation or re-infection by different species or strains of the parasite.

**Conclusions:**

Wide promotion of sanitary education is strongly justified in children and adolescents in *Toxocara* endemic areas in order to reduce the potential risk of primary invasion or re-infection with the parasites, which can lead to a severe course or progression of the disease. A long-term clinical follow-up and more intensive anti-parasitic treatment is recommended in patients with subclinical and overt forms of toxocarosis to prevent later reactivation of the migrating larvae in tissues.

## Introduction

Toxocarosis is a helminth infection of humans caused by the dog or cat roundworm - *Toxocara canis* or *Toxocara cati*, respectively. A man is a paratenic host of *Toxocara* spp., who can become infected by accidentally ingesting invasive eggs in contaminated soil. The eggs hatch in the stomach and infective larvae undergo a somatic migration to a wide variety of tissues (liver, lungs, brain, eyes) causing local inflammatory and allergic reactions, but fail to mature into adult forms. The presence of migrating larvae within the tissues contributes to pathology, the severity of which is dependent upon the intensity of infection and the location of the larval forms.

Ocular larva migrans syndrome (OLM) is a localized manifestation of a *Toxocara canis* or *Toxocara cati* eye infection, usually caused by a single second-stage larva. Despite the low intensity of invasion and unilateral location of the migrating parasite, infection may cause severe inflammation and progressive ocular damage, leading to retinal detachment, cataract formation, endophthalmitis, strabismus and blindness, usually observed in older children and adolescents below the age of sixteen [1,2]. In some geographic areas, e.g. in the state of Alabama in the United States, OLM is considered an endemic disease that occurs with an incidence rate of 1 per 100,000 persons in the general population or 1 per 1000 patients at eye clinics [1]. Eye disease results from the immunopathological response to the presence of migrating *Toxocara species* larvae and their excretory-secretory antigens (TES), which are located in a closed ocular compartment, usually in the retina, and are sporadically accompanied by systemic signs of the infection. The eosinophil count can sporadically increase to more than 400 cells per μl, and a titre of IgG antibody specific for *Toxocara species* is of low diagnostic value, because a majority of OLM cases do not result in a general stimulation of the immunological system and specific IgG can show low or even undetectable values in the peripheral blood [3]. Similarly, the levels of *Toxocara*-specific IgG in the peripheral blood are significantly much higher in patients with the systemic disease in comparison to OLM cases.

Visceral larva migrans syndrome (VLM), which is characterized by fever, hepatosplenomegaly, abdominal pain, weight loss, chronic cough and bronchospasm, usually occurs in younger children of up to 7 years of age, and can be easily diagnosed on the basis of peripheral hypereosinophilia, leucocytosis, hyper-gammaglobulinaemia, and positive *Toxocara species* serology [1,4,5]. The generalized form of the infection is caused by the ingestion of a high dose of the invasive eggs of the parasite, resulting in a more intense multiorgan infection compared to a localized OLM syndrome [6]. Circulating larval antigens of *Toxocara species* stimulate Th0 lymphocytes to develop into active Th2 cells, which initiate the synthesis of interleukin-4 (IL-4) and interleukin-5 (IL-5), responsible for the production of total and specific anti-parasitic immunoglobulin E antibodies by plasma cells, and accelerated maturation of eosinophils, respectively. The ability of the larvae to survive and migrate in tissues of their hosts for months or even years provokes a stable stimulation of Th2 lymphocytes and a persistent production of IgE for a long time [4].

We are reporting three atypical cases of severe toxocarosis in school-age children living in high-risk areas. The patients had a documented exposure to *Toxocara species* eggs and a daily contact with domestic animals in a rural environment. We detected intraocular synthesis of specific antibodies to *Toxocara canis* in the anterior chamber fluids, and estimated tissue eosinophilia and the local stimulation of allergic reactions in the ocular compartment.

The levels of anti-*Toxocara* immunoglobulin G antibody in serum samples were measured by a classic ELISA test (Bordier Affinity Products, Crissier, Switzerland). Comparative immunological profiles of *Toxocara* spp.-specific IgG antibodies in aqueous humour and serum samples were measured by the Western blot assay (LDBIO Diagnostics, Lyon, France). The presence of IgG bands against *Toxocara*-specific antigens of 24-35 kDa of different antigenic specificity or synthesised in a higher concentration in anterior chamber fluids when compared with serum samples, indicated local immune response in ocular toxocarosis.

Serum concentrations of the soluble subunit of human CD23 molecule (sCD23), which is a 45 kDa protein found on the surface of eosinophils, macrophages and some cytotoxic T cells, were estimated using a reverse ELISA (Bender MedSystems, Vienna, Austria). CD23 cytokine plays a significant role in the regulation of IgE synthesis and can evoke immune response to parasitic infections caused by helminths. For this reason, the sCD23 molecule was evaluated as a marker of local tissue eosinophilia in cases of toxocarosis.

The total IgE levels in the anterior chamber fluids and serum samples were tested using classic ELISA (IBL, Hamburg, Germany). The aqueous humour/serum sample IgE coefficient was calculated in the patients to evaluate the intensity of the local intraocular allergic or hypersensitivity reactions to the presence of migrating *Toxocara species* larvae.

## Case presentation

### Case report 1

**Figure 1.**
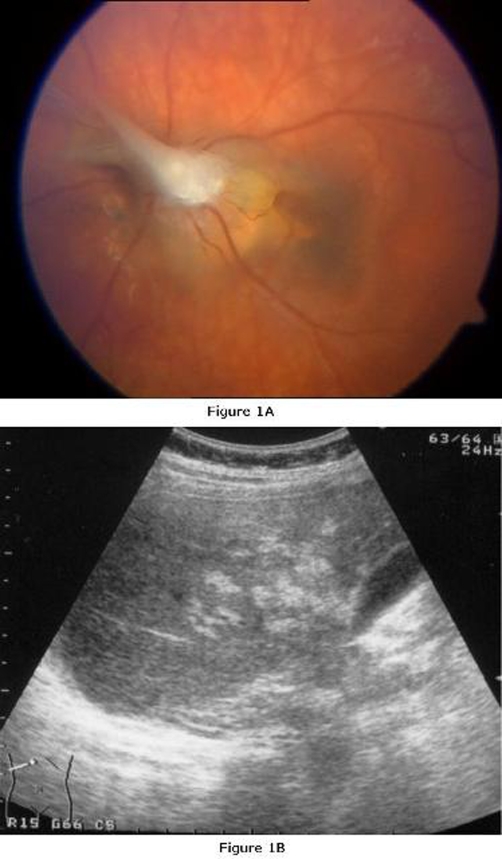
**(A)** Sickle-shaped epiretinal membrane and inactive post-inflammatory retinal scar of the left eye in the 8-year-old child infected with *T. canis,* as visualized using direct ophthalmoscopy. **(B)** Multiple hepatic granulomas in the 8-year-old child infected with *T. canis*. An ultrasound of the abdomen is shown.

An 8-year-old Polish boy came to our Clinical Department with a 7-month history of visual problems and divergent strabismus in his left eye. He was referred to the clinic by a school nurse because of reading problems. The patient lived in a rural area and had frequent contact with young dogs and cats. On admission, direct ophthalmoscopy of the left eye showed an epiretinal sickle-shaped membrane growing from the disc of the optic nerve to the periphery of the retina which was surrounded by an inactive chorioretinal scar (Figure 1A). An examination of anterior eye segment showed vitreous inflammation. The visual acuity of the affected eye was 3/50 when evaluated using the Snellen chart. No abnormalities were found in the right eye. The direct eosinophil count in peripheral blood was slightly elevated to 500 cells per μl (normal range: 0-440 eosinophils/μl) without leucocytosis (WBC count was 8.2 × 103/μl). The serological examination showed a high titre of specific anti-*T. canis* IgG antibody in a serum sample measured by ELISA (O.D. = 2.051). Ultrasonography of the abdomen revealed multiple hyperechogenic lesions consistent with hepatic granulomas (Figure 1B) without an increase in liver enzyme activity in the peripheral blood (ALT) 17 IU/ml and (AST) 28 IU/l). The anterior chamber of the affected eye was punctured in order to test for the local synthesis of anti-*T. canis* IgG in the aqueous humour. The Western blot assay confirmed ocular infection with *Toxocara* spp. by detecting four specific bands of IgG directed against specific antigens of 24-35 kDa found at a higher concentration than in peripheral blood. The level of sCD23 cytokine in the serum sample was elevated (52 U/mL). The child was treated with two 10-days courses of albendazole at a daily dose of 15 mg/kg body weight in two divided doses. Two months later, when he was examined in the outpatient clinic, his left visual acuity improved to 20/100.

### Case report 2

**Figure 2.**
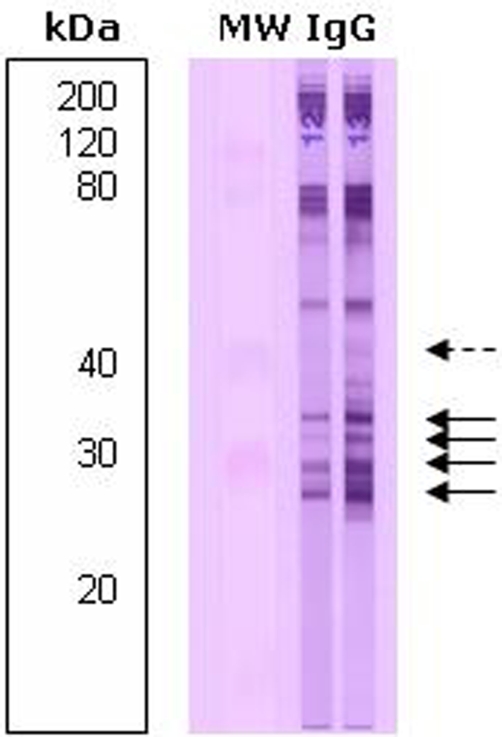
Comparative immunological profiles analysis of aqueous humour and serum samples using the Western blot assay in a 14-year-old patient with ocular and visceral toxocarosis. Arrows indicate locally synthesized bands of *Toxocara* spp.-specific IgG in the intraocular fluid. 12 - serum sample, 13 - aqueous humour sample.

A 14-year-old adolescent Polish boy was sent to our Clinic by an ophthalmologist for confirmation of ocular larva migrans syndrome. The patient complained of a sudden loss of visual acuity 6 years ago and a progressive visual loss during the last 4 years. An epidemiological interview confirmed a direct contact with dog pets that were not regularly dewormed and a frequent contact with the soil. On admission, unilateral blindness with divergent strabismus of his right eye, with co-existing non-specific abdominal pain, was diagnosed. The examination of the eye fundus showed two subretinal granulomas, one located in the macula of the right eye and another one in the periphery of the retina with fibrous epiretinal membranes. The anterior segment was clear and the intraocular pressure was normal. Brain magnetic resonance imaging of the infected eye showed a hyperdense space-occupying lesion in the posterior pole imitating retinoblastoma with superficial calcifications detected by an ultrasound, without signs of retinal detachment or orbit infiltration, and a similar satellite tumour-like lesion located in the periphery. An ultrasound of the abdomen revealed moderate hepatosplenomegaly (the vertical dimension of the right lobe was 15.5 cm). Blood tests showed significant hypereosinophilia of 1.2 × 103 per μl (total WBC count was 8.3 × 103/µl) and an equivocal level of anti-*T. canis* specific IgG antibody with an O.D. value of 0.764. The serum concentration of non-specific IgE was highly elevated (563 IU/ml). No signs of atopic diseases were found. Alanine transaminase and aspartate transaminase values were normal (8 and 21 IU/l respectively). The comparative immunological profiles confirmed local anti-*Toxocara* IgG antibody production in the aqueous humour (Figure 2). Three 10-days courses of anti-parasitic treatment with albendazole at a dose of 15 mg/kg body weight/day were prescribed. Non- progression of the eye lesions with a normalization of blood eosinophilia and total IgE were observed during the 16 months follow-up.

### Case report 3

**Figure 3.**
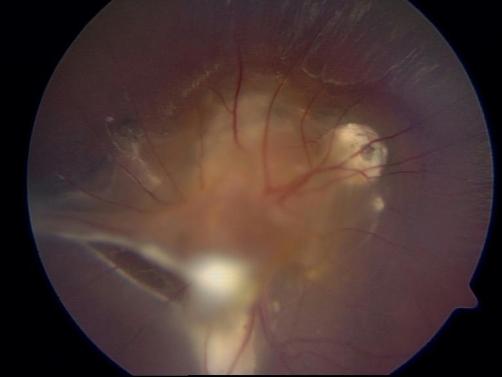
Central and peripheric subretinal granulomas with two fibrous traction bands proliferating to the periphery, detected by an examination of the eye fundus in a 16-year-old adolescent with ocular larva migrans syndrome.

A 16-year-old Polish adolescent was admitted to the Department with severe vision impairment and divergent strabismus of his left eye that had been present since the pre-school period. In his epidemiological anamnesis there was a history of close contact with dogs and cats, and geophagia during early childhood. Ophthalmological consultation showed a large subretinal granuloma in the macula and optic disc of the left eye, and two additional granulomas in a far peripheral location with fibrous traction bands proliferating to the periphery, macular scarring consistent with previous retinitis, and vitritis complicated by a cataract (Figure 3). The visual acuity in the affected eye was limited to hand movements and 5/5 in the other eye. The patient complained of chronic abdominal pain, and physical examination showed obesity (BMI = 30.5). There was no peripheral eosinophilia (200 eosinophils/µl). Routine blood serology was significantly positive for *T. canis-*specific IgG antibody (O.D. = 1.365). Additionally, Western blotting showed local synthesis of specific IgG antibody bands in the anterior chamber fluid from his left eye. The concentrations of non-specific IgE in the aqueous humour and serum samples were similar, with values of 60 and 67.5 IU/ml (IgE coefficient was 0.89) respectively. The patient received two 10-days courses of anthelmintic therapy with albendazole and local administration of steroids. On discharge the visual acuity in the affected eye improved and was 0.5/50.

## Discussion

Toxocarosis is a common parasitic infection, which may present with various clinical manifestations in children and young people. Children are more susceptible to infection because of closer contact with puppies and kittens, lack of compliance with general hygienic measures, and have more opportunities to be infected by contact with contaminated soil or sand in public parks and playgrounds. *Toxocara* infection may not be diagnosed during a routine paediatric examination due to its unusual symptomatology resembling other paediatric diseases, such as retinoblastoma, bronchitis, asthma, and atopic disorders [5]. The clinical spectrum of toxocarosis reflects various manifestations depending on the internal organs infected by the migrating worms and the intensity of infection. The pattern varies in severity, and can include (i) asymptomatic infection detected by positive serology alone, which is the most common case; (ii) visceral and incomplete visceral larva migrans syndrome (VLM); (iii) localized form of ocular larva migrans syndrome (OLM) or neurotoxocarosis; and (iv) occult or covert toxocarosis with non-specific symptoms or signs resulting from an immunopathogenic response to circulating excretory-secretory *Toxocara species* antigens. Isolated findings include acute bronchitis, pneumonitis with or without Löffler’s syndrome, wheezing, lymphadenitis, chronic urticaria, pruritus, myositis and reactive arthritis [7,8]. The covert form of toxocarosis is usually documented retrospectively by diminished or total regression of non-specific clinical symptoms or signs after the implementation of anthelmintic treatment. According to some practitioners, there is a high risk of the transformation of untreated covert toxocarosis to symptomatic neurotoxocarosis or OLM syndrome in the absence of a specific anti-parasitic therapy [9,10].

Symptoms of a classic VLM syndrome may be due to host inflammatory response to repeated passages of migrating larvae through the viscera, whereas OLM occurs in individuals who have not been previously immunized by *Toxocara* parasites [6]. The clinical differences may also be due to the number of infective larvae ingested and the difference in the migratory behaviour of *T. canis* and *T. cati* parasites, which is relative to variations in the host inflammatory response. A highly infective dose of *Toxocara* spp. eggs ingested is responsible for the development of typical VLM syndrome with symptomatic hepatic and pulmonary involvement soon after invasion [6,9]. In contrast, OLM is usually the consequence of colonization of the eye by a single migrating *Toxocara species* larva [1,9]. Infection with less number of infective *Toxocara* eggs is insufficient to stimulate a significant rise in eosinophils and specific antibody levels in the peripheral blood. As a result, larvae migrate unimpeded through the liver and lungs and induce minimal tissue response or clinical signs. These few larvae may enter the circulation and migrate randomly resulting in occasional eye infection. In patients with classical OLM syndrome, the generalized signs of visceral damage, which occur in VLM, are usually not observed. Some authors suggest the possibility that there might be some strains of *Toxocara* with specific tropisms to particular tissues or systems of the infected host [3].

*Toxocara* infections have been classified clinically as OLM and VLM largely because the ocular disease has a tendency to occur in the absence of systemic involvement and vice versa [3]. Cases of OLM are infrequently accompanied by peripheral hypereosinophilia, and the co-existence of ocular toxocarosis with visceral clinical syndrome in the same patient has rarely been reported.

Lalosevic et al. reported a case of a 6-year-old child with left side strabismus, granulomatous chorioretinitis and a high level of eosinophils in the peripheral blood (24%) but visceral signs of generalized *Toxocara* infection were not seen [11]. In the study by Altcheh et al. [12] absolute eosinophil count was statistically less elevated in patients with a classic OLM syndrome than in a group of VLM patients or asymptomatic individuals with *Toxocara*-positive serology alone. Moreover, there was no hepatic or pulmonary involvement in children with a localized form of ocular infection, and similarly VLM cases have not demonstrated any ophthalmological problems. In case of a series of ocular toxocarosis in adults published by Park et al. [13], there were no hepatobiliary symptoms or increase in liver enzymes. One of the patients, who complained of decreased visual acuity with tractional retinal detachment and inflammatory exudates had highly elevated titres of total and anti-*Toxocara* specific immunoglobulin E antibodies (3116 IU/ml and 34,000 TU, respectively), and hypereosinophilia in the peripheral blood (3,340 eosinophils per µl) [17]. For this reason, imaging diagnosis of the abdomen or lungs is sporadically made in patients with typical ocular toxocarosis presented in form of unilateral posterior uveitis, subretinal granuloma, strabismus and leukocoria. The lack of a profound clinical examination in patients with OLM syndrome may be the main cause of the misdiagnosis of the co-existence of more severe ocular and visceral forms of *Toxocara species* infection, which differ with respect to the clinical picture, treatment recommendations and patients’ prognosis. Specific anti-parasitic therapy should be more intensified or prolonged in severe cases with confirmed ocular or mixed forms of *Toxocara species* infection. We propose 2-3 courses of a 10-day anti-parasitic treatment with albendazole at a dose of 15 mg/kg body weight/day, given at 4-weekly intervals, in order to kill most of the migrating larvae and reduce the risk of a delayed re-activation. On the contrary, the classic VLM syndrome in children and adults may be traditionally treated with a short 5-day course of albendazole, with good tolerance and clinical efficacy. Repeated cycles of anthelmintic treatment should only be considered in some patients from high risk areas with signs of re-infection, resulting from constant contact with contaminated soil and/or domestic animals (professional hazard), as well as unhealthy dietary, and hygienic habits like geophagia, onychophagia, placing of fingers in the mouth or eating raw animal livers.

Maffrand et al. [14] described a rare case of congenital toxocarosis in a neonate born prematurely by a *Toxocara*-positive mother at 32 weeks of pregnancy, with extensive eosinophilia of 30% (4.560 eosinophils per µl), leucocytosis, signs of respiratory distress, and intraocular tumour-like mass imitating retinoblastoma. After 2-weeks of anthelmintic treatment with tiabendazole, a resolution of the hyperdense retinal lesion, which contained the migrating larva of *Toxocara* was observed, and the eosinophil count in the peripheral blood was diminished to up to 6% (700 cells per µl). The exceptionally high degree of parasitic infection in the mother, and possible immunosupression of the immature newborn resulting in hypogammaglobulinaemia, low Apgar scores, low birth weight and retinopathy of prematurity could be probably related to a multiorgan expression of the systemic toxocarosis in the neonate [14].

The three patients presented in this study, all demonstrated unusual clinical forms of *Toxocara species* infection. In patients 1 and 2, a rare association of localized OLM and systemic VLM syndromes with splenomegaly or hepatomegaly with hepatic granulomas, as well as peripheral blood hypereosinophilia were documented. In the first case, a generalized *Toxocara species* infection was additionally confirmed by the detection of sCD23 cytokine, which is a soluble subunit of the eosinophil receptor for IgE and may be a valuable immunological marker of tissue eosinophilia. So far, the testing of sCD23 in parasitic infections is not widely proposed. Elevated concentrations of sCD23 were found in research studies in patients with chronic lymphocytic leukaemia, hairy cell leukaemia, hyper IgE syndrome and after bone marrow transplantations [15,17]. In the second patient, a highly elevated concentration of non-specific immunoglobulin E antibody indicated systemic involvement with signs of hypersensitivity or allergic reactions induced by circulating TES antigens of the parasites. In the third case, equivocal titres of non-specific IgE found not only in the serum but also in the aqueous humour sample suggested a generalized response to the parasitic infection.

The parasitic aetiology of subretinal granulomas was confirmed by the presence of intraocular synthesis of *Toxocara species*-specific IgG antibodies in the anterior chamber fluid of the infected eyes of all three patients, including one with uncertain peripheral blood serology (patient 2). In clinical practice, ocular toxocarosis is rarely confirmed by testing the local production of specific antibodies in aqueous or vitreous fluid, and a final diagnosis is generally made on the basis of typical clinical signs, with co-existing positive serology from peripheral blood [18]. The patients presented in the study all have unquestionable eye disease caused by the *Toxocara* parasites diagnosed by comparative immunological profiles shown by western blotting.

Abdominal pain observed in patients 2 and 3 was consistent with the visceral form of symptomatic toxocarosis. Some authors suggest a higher prevalence of abdominal pain in *Toxocara*-seropositive children, especially in those with more severe infection documented by strongly elevated titres of specific antibodies [4]. Additionally, in these two children from high-risk rural areas, the clinical pattern of the multifocal eye disease may probably be due to the late re-activation of quiescent larval forms of *Toxocara species* or a possible re-infection during a repeat exposure to a *Toxocara species*-contaminated environment. In patient 2, who had a low level of *Toxocara specie*s-specific serum antibody, a subretinal granuloma appeared in the periphery of the previously affected eye 2 years after the initial infection. The possibility of the activation of a dormant *Toxocara species* larva existing in a “hypobiosis” (lower metabolism) condition cannot be excluded. Despite the acquired immunity demonstrated by a high serum titre of specific IgG in the third patient, a severe infection caused by multiple larval forms of *Toxocara species* was observed. The ocular compartment is known to be an area of a low immunological response against parasitic infections due to the lack of infiltration of inflammatory cells and cytokines. In this third case, the developed semi-immunity was not sufficient to protect the adolescent against the new infection of the eye.

The double clinical picture may suggest a mixed infection with two different strains of *Toxocara species,* characterized by various pathogenic features or even a secondary infection with another strain of the same parasite species. The possibility of concomitant *T. canis* and *T. cati* infections is also a possibility. From clinical observation, there was no induction of stable protective immunity during the primary *Toxocara species* infection and the susceptibility to a re-invasion in case of high degree of contaminations.

The cases presented in the study illustrate the need for prompt and intensive anti-parasitic treatment in asymptomatic or subclinical *Toxocara*-seropositive children in order to diminish the risk of eye damage. Changes in the behaviour and/or environment of infected children and regular deworming of domestic animals can be beneficial to reduce or eliminate the risk of a continued exposure to *Toxocara* eggs. A prolonged clinical observation is crucial in cases infected with *Toxocara species*, in order to prevent the late re-activation of quiescent parasites located deep within human tissues.

## Conclusions

The co-existence of ocular larva migrans syndrome and visceral signs of *Toxocara* spp. infection in children and adolescents is a rare clinical form of toxocarosis but cannot be neglected, because of the more severe clinical picture with irreversible sequelae.

A more effective sanitary education on *Toxocara* and a more healthy life style is needed in school-age children in highly endemic areas, in order to reduce the potential risk of infection or re-infection, which may lead to severe incidences of toxocarosis and the progression of the disease.

Prolonged anti-parasitic treatment is recommended in patients with asymptomatic or subclinical forms of *Toxocara* infection in order to prevent the late reactivation of the migrating larvae in tissues.

## List of abbreviations

ALT: Alanine transaminase
AST: Aspartate transaminase
BMI: Body mass index
ELISA: Enzyme-Linked Immunosorbent Assay
Ig: Immunoglobulin
IU: International unit
MW: Molecular weights
O.D.: Optical density
OLM: Ocular larva migrans
SD: Standard deviation
TU: *Toxocara* unit
TES: *Toxocara* excretory-secretory antigens
VLM: Visceral larva migrans
WBC: White blood cells
